# Evaluation of the efficiency of peptide receptor radionuclide therapy in patients with metastatic prostate carcinoma: A protocol for systematic review and meta-analysis

**DOI:** 10.1097/MD.0000000000025612

**Published:** 2021-04-30

**Authors:** Gang Liu, Tang Tang, Xiao-Peng Liu, Zhong-Hua Zhou, Feng-Jiao Li

**Affiliations:** a Department of Nuclear Medicine; b Department of Gynaecology, Zhuhai Hospital of Integrated Traditional Chinese and Western Medicine, Zhuhai, Guangdong, P.R. China.

**Keywords:** efficiency, meta, peptide receptor radionuclide therapy, prostate carcinoma

## Abstract

**Background:**

Metastatic prostate carcinoma has poor prognoses with a median survival period ranging from 2 to 5 years with existing therapeutic challenges. Currently, peptide receptor radionuclide therapy is permitted as a treatment method for metastatic prostate carcinoma patients. Therefore, it is crucial to comprehend the efficiency and safety of peptide receptor radionuclide therapy among this patient population. This study aims to analyse the efficacy of peptide receptor radionuclide therapy when used to treat metastatic prostate carcinoma patients.

**Methods:**

This research will perform a methodological search in the following electronic databases to find related randomized controlled trials: Cochrane Library, EMBASE, PubMed, Web of Science, Scopus, China National Knowledge Infrastructure (CNKI), WanFang database, and Chinese BioMedical Literature. All the databases are searched from their inauguration till November 2020. Two independent authors will screen and select literature for review. The two authors will independently utilize the Cochrane Risk of Bias tool to assess the bias risk in studies. This study also plans to conduct subgroup and sensitivity analyses to evaluate the robustness in the results. Statistical analyses will be conducted with the RevMan 5.3 software.

**Results:**

A high-quality synthesis of existing evidence related to peptide receptor radionuclide therapy in the treatment of metastatic prostate carcinoma will be presented in this study.

**Conclusion:**

Our findings will provide evidence to judge whether peptide receptor radionuclide treatment is efficient for metastatic prostate carcinoma patients.

**Ethics and dissemination:**

An ethics approval is not required because the data of the present study are primarily obtained from published studies.

OSF registration number: December 1, 2020.osf.io/3psx7. (https://osf.io/3psx7/).

## 1 Introduction

Based on the GLOBOCAN2018 estimates, around 1.3 million fresh cancer patients and 360,000 fatalities were reported worldwide.^[1]^ For males, prostate cancer causes the second highest cancer-related mortality rate and the most common malignancy of the male genitourinary system across the world.^[2]^ Most patients are diagnosed with metastatic prostate carcinoma during the initial diagnosis. Several studies have shown that almost every patient ultimately develop castration-resistant prostate carcinoma after treatment.^[3,4]^ Prostate carcinoma patients treated at early stages have a positive prognosis with a 5-year overall survival rate reaching 99%. Despite significant developments in the treatment for prostate carcinoma during the past decade, since the median overall survival remains poor, patients with prostate carcinoma prognosis are largely unsatisfied.

Techniques based on androgen ablation treatment is prevalent for recurring illnesses, metastatic disease, or advanced-stage prostate cancer. Normal androgen deficiency therapy and fresh androgen axis drugs are usually quite tolerable and can stabilize metastatic hormone-sensitive prostate cancers for long periods.^[5–7]^ Identifying possible treatment markers in metastatic/advanced prostate cancer and androgen-independent condition is crucial for enhancing diagnosis and therapeutic strategies. Perfect markers for prostate cancer therapy would entail structures that are entirely expressed in standard prostate tissue, these have elevated expression in metastatic disease, and therapeutic modalities at the cell surface have access to these structures.^[8–10]^ After previously promising results with 177Lu-labeled prostate-specific membrane antigen radioligand treatment marks prostate-specific membrane antigen, containing high expression levels on the surface of prostate cancer cells.^[11]^ Following several lines of therapy, prostate-specific membrane antigen expression in metastases remain high, allowing systemic radioligand therapy via repeated intravenous applications of the radioligand.^[12,13]^ Therefore, devising effective therapeutic strategies for metastatic prostate cancer is a challenge that is of epidemiological significance among aging populaces. Peptide receptor radionuclide therapy (PRRT) is now allowed for patients with metastatic prostate carcinoma. Therefore, it is crucial to comprehend the effectiveness and security of PRRT in this patient populace. Resultantly, a systematic review and meta-analysis are performed to assess the efficacy of peptide receptor radionuclide treatment in metastatic prostate carcinoma patients.

## 2 Objectives

The objective of the present study is to present a protocol study to evaluate the efficiency of PRRT when treating metastatic prostate carcinoma patients.

## 3 Methods

### 3.1 Study registration and design

This systematic review protocol has been registered on OSF 10.17605/OSF.IO/3PSX7 and designed using the rules defined by Preferred Reporting Items for Systematic review and Meta-Analysis Protocol (PRISMA-P) statement.^[14]^

### 3.2 Eligibility criteria for included studies

#### 3.2.1 Types of participants (P)

The present study will include participants confirmed with histological diagnosis of prostate and radiologic evidence of metastases as determined by computer-aided tomography, magnetic resonance imaging technique, or positron emission tomography with or without bone scans. Resections were not placed on age and treatment strategy.

#### 3.2.2 Types of interventions (I) and comparison (C)

The experimental group received PRRT, and the comparator group will be administered a different course of treatment (i.e., luteinizing hormone-secreting hormone agonist, antagonist, anti-androgen receptors, combination of luteinizing hormone-releasing hormone agonist plus antiandrogen, or bilateral orchiectomy).

#### 3.2.3 Types of outcome measures (O)

The major outcome for the present study is time to death from any cause. The minor outcomes for the present study include quality of life, discontinued treatment because of adverse events, time to disease progression, and time to death due to prostate cancer.

#### 3.2.4 Types of studies (S)

This study will include randomized controlled trials, regardless of blinding, allocation concealment, and their publication status.

### 3.3 Eligibility criteria for identification of studies

#### 3.3.1 Search strategy

A methodological search is done in the following electronic databases to source randomized controlled trials: PubMed, EMBASE, Cochrane Library, Web of Science, Scopus, China National Knowledge Infrastructure (CNKI), WanFang database, and Chinese BioMedical Literature. All databases are search from their inauguration till November 2020. The search does not include any restrictions on the language of publication. The key search terms were MESH terms and free text words as follows: (“metastatic prostate carcinoma∗” OR “metastatic prostate cancer∗” OR “metastatic prostate tumour∗” OR “metastatic prostate tumor∗” OR “metastatic prostate neoplasm∗”) AND (Lutetium [MeSH] OR lutetium∗ OR “peptide receptor radionuclide therapy” OR PRRT).

#### 3.3.2 Search other resources

Attempts will also be made to source other potentially suitable studies or secondary publications by scrutinizing reviews, meta-analysis, and reference lists of selected studies. Moreover, additional studies are identified by contacting study authors.

### 3.4 Data collection and analysis

#### 3.4.1 Study selection and data extraction

**Figure 1:**
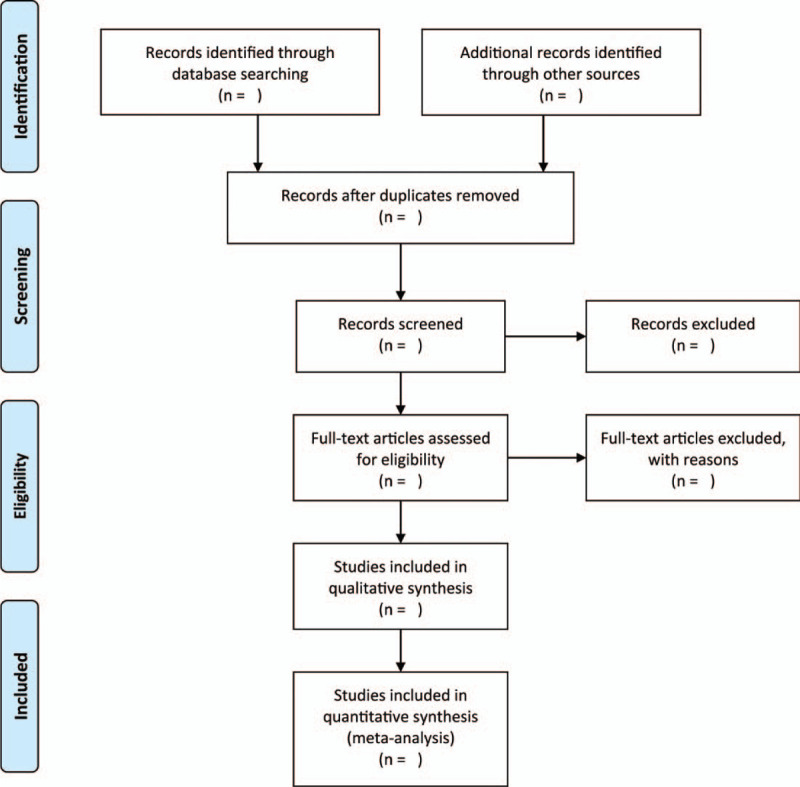
The research flowchart.

This study utilizes EndNote X9 to locate and eradicate possible duplicates. Two independent authors will scan abstracts/titles of selected studies to evaluate studies that are suitable for further assessment. Two independent authors will examine all relevant records as full-texts and separate studies under included or excluded studies. It is planned to resolve any disagreement through consensus by a third author. The following baseline characteristics are extracted from the included studies by the two independent authors: first author, published year, study design, study settings and country, mean age, number of participants, intervention method, and relevant outcomes. The selection process will be shown in Figure 1.

#### 3.4.2 Assessment of risk of bias in included studies

Two independent authors will evaluate the bias risk in each included study using Cochrane Collaboration's “Risk of bias” assessment tool.^[15]^ Any disagreement will be resolved via consultation with a third author.

#### 3.4.3 Measures of treatment effects

Dichotomous data will be expressed as the risk ratio together with 95% confidence intervals. Continuous data will be expressed as the mean difference or standardized mean difference together with 95% confidence interval.

#### 3.4.4 Dealing with missing data

In the case of any missing data, it is planned to contact the corresponding author to collect the data if available. If recovering sufficient data is unsuccessful, we will inspect study reports with missing data and report the reasons for the lack of data.

#### 3.4.5 Assessment of heterogeneity

Statistical heterogeneity will be evaluated using the *I*^2^ statistic. We plan to consider a level of heterogeneity more than 50% as significant heterogeneity; in which case the random-effects model will be used to pool the data.

#### 3.4.6 Assessment of reporting biases

Attempts will be made to collect study protocols to evaluate for selective outcome reports.

#### 3.4.7 Sensitivity analysis

If applicable, we will perform a sensitivity analysis to explore the reliability and generalization of our results.

## 4 Discussion

Recently, there has been an increase in randomized controlled trials of PRRT for treating metastatic prostate carcinoma. Although several published studies have concluded that applying PRRT holds a substantial position for the treatment of metastatic prostate carcinoma. However, the effectiveness of PRRT in metastatic prostate carcinoma patients is yet to be established conclusively. Therefore, this systematic review and meta-analysis aim to evaluate the efficacy of PRRT in metastatic prostate carcinoma patients. It is hoped that the findings will offer clinicians with the basis for PRRT of metastatic prostate carcinoma.

## Author contributions

**Conceptualization:** Gang Liu, Tang Tang, Xiao-Peng Liu.

**Data curation:** Gang Liu, Tang Tang, fengjiao li.

**Formal analysis:** Gang Liu, Xiao-Peng Liu.

**Funding acquisition:** Tang Tang.

**Investigation:** Gang Liu.

**Methodology:** Xiao-Peng Liu, Zhong-Hua Zhou, Feng-Jiao Li.

**Project administration:** Tang Tang, Xiao-Peng Liu, Zhong-Hua Zhou.

**Resources:** Zhong-Hua Zhou, Feng-Jiao Li.

**Software:** Gang Liu, Xiao-Peng Liu, Feng-Jiao Li.

**Supervision:** Tang Tang.

**Validation:** Tang Tang, Zhong-Hua Zhou.

**Visualization:** Gang Liu, Xiao-Peng Liu, Feng-Jiao Li.

**Writing – original draft:** Gang Liu, Feng-Jiao Li.

**Writing – review & editing:** Xiao-Peng Liu, Zhong-Hua Zhou, Feng-Jiao Li.
